# In vitro modeling of the neurovascular unit: advances in the field

**DOI:** 10.1186/s12987-020-00183-7

**Published:** 2020-03-16

**Authors:** Aditya Bhalerao, Farzane Sivandzade, Sabrina Rahman Archie, Ekram Ahmed Chowdhury, Behnam Noorani, Luca Cucullo

**Affiliations:** 1 Department of Pharmaceutical Sciences, Jerry H. Hodge School of Pharmacy, Texas Tech University Health Sciences Center, 1300 S. Coulter Street, Amarillo, TX 79106 USA; 2 Center for Blood-Brain Barrier Research, Texas Tech University Health Sciences Center, Amarillo, TX 79106 USA

**Keywords:** Blood–brain barrier, Neurovascular unit, 3D models, Microfluidics, Organoids, Brain microvasculature

## Abstract

The blood–brain barrier (BBB) is a fundamental component of the central nervous system. Its functional and structural integrity is vital in maintaining the homeostasis of the brain microenvironment. On the other hand, the BBB is also a major hindering obstacle for the delivery of effective therapies to treat disorders of the Central Nervous System (CNS). Over time, various model systems have been established to simulate the complexities of the BBB. The development of realistic in vitro BBB models that accurately mimic the physiological characteristics of the brain microcapillaries in situ is of fundamental importance not only in CNS drug discovery but also in translational research. Successful modeling of the Neurovascular Unit (NVU) would provide an invaluable tool that would aid in dissecting out the pathological factors, mechanisms of action, and corresponding targets prodromal to the onset of CNS disorders. The field of BBB in vitro modeling has seen many fundamental changes in the last few years with the introduction of novel tools and methods to improve existing models and enable new ones. The development of CNS organoids, organ-on-chip, spheroids, 3D printed microfluidics, and other innovative technologies have the potential to advance the field of BBB and NVU modeling. Therefore, in this review, summarize the advances and progress in the design and application of functional in vitro BBB platforms with a focus on rapidly advancing technologies.

## Background: structure and functions of the BBB

The BBB consists of highly specialized vascular endothelial cells (EC) lining the brain microvessels in juxtaposition with closely associated pericytes [1], extracellular matrix components, and astrocytic end-feet processes [2]. Along with other cells such as neurons and microglia, this cellular milieu modulates the BBB properties, supports its viability and functions [3]. At the brain microvascular level, the BBB functions as a highly dynamic and active interface between the systemic circulation and the CNS. The BBB maintains a stable brain environment to protect the CNS from unsolicited cells, bacteria, viruses, and potentially harmful substances (either endogenous or exogenous) apart from protecting against systemic fluctuations. The BBB also regulates the transport of essential molecules and nutrients necessary to maintain the optimal CNS environment and support neuronal activities [4]. The BBB responds to many physiological and pathological cues, including rheological changes [5], inflammatory stimuli, oxidative stress [6], diabetes, and hypercholesterolemia [7–10], acute brain injury [3], etc. The intrinsically unique and utmost complex functional interaction between the BBB endothelium and the surrounding cellular milieu (including astrocytes, pericytes, neurons, microglia, myocytes as well as specialized cellular compartments such as endothelial glycocalyx [11, 12] has been termed “neurovascular unit (NVU)” [2, 13]. In addition, the basement membrane, which exerts essential functions in cellular support and signal transduction, arises from the extracellular matrix (ECM) proteins secreted by ECs and astrocytes [14, 15].

**Fig. 1:**
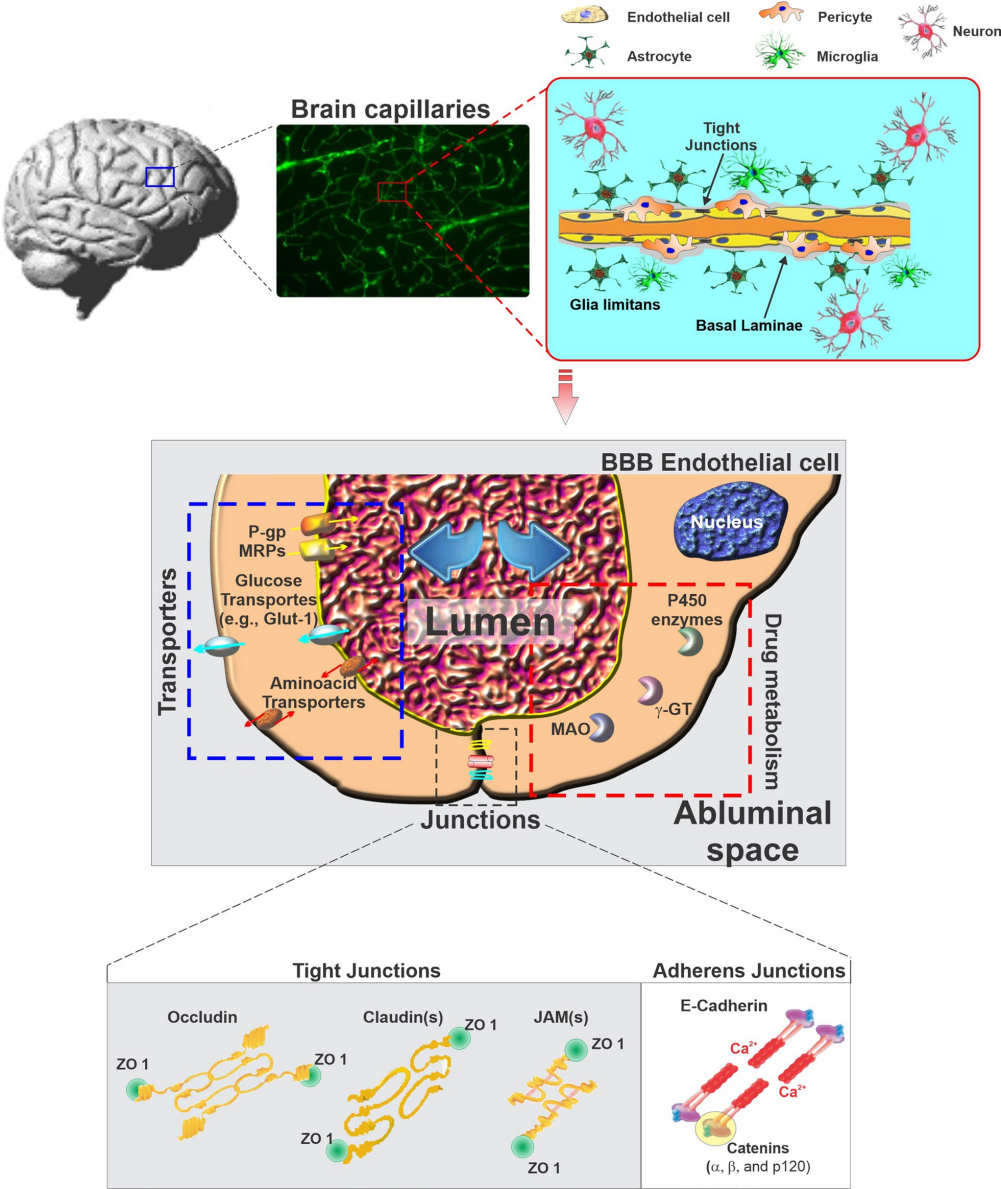
Schematic view of a typical brain microcapillary. Note that the passage of substances across the BBB endothelium is controlled by a multimodal barrier system; (1) gating barrier (tight junctions) which prevents paracellular diffusion of polar molecules. Note that the adherens junction play the critical role of keeping the cell membrane of adjacent endothelial cells close together, thus allowing for the formation of the tight junctional bindings; (2) transport barrier which includes number of active efflux systems (P-gp, MRPs, etc.) with affinity for lipophilic substances; (3) metabolic/enzymatic barrier (cytochrome P450 enzymes, MAO, etc.) which catalyze the oxidation/metabolism of organic substrates including xenobiotic substances such as drugs and other potentially toxic chemicals

Unlike their peripheral counterparts, the BBB endothelial cells are characterized by limited pinocytosis, the relative absence of fenestrations, and asymmetrical expression (lumen versus albumen) of trans-membrane transport and efflux systems regulating the traffic of substances between the blood and the brain parenchyma [16]. Transmembrane inter-endothelial TJ proteins (e.g., occludin, claudins, etc.) restrict the paracellular flux of ions and hydrophilic solutes across the BBB [17] resulting in high electrical resistance with readings ≥ 1800 Ω cm^2^; measured in situ in rats [18]. TJs also work as a “fence” that limits the free movement of lipids and proteins within the plasma membrane between the apical and the basal surface. Thus, water-soluble nutrients and other biologically vital substances (including amino acids, d-glucose, mono-carboxylic acids, etc. [16]. are delivered into the brain by specialized carrier-mediated transport systems [16] (see Fig. 1).

Furthermore, other prominent protein families, such as adherens junctions (AJ) and gap junctions (GJ), play significant roles in intercellular adhesion and communication, respectively, and are integral to BBB tightness [11]. In addition to the TJs, the BBB endothelium expresses a host of efflux transporters (including P-glycoprotein—P-gp [19], breast cancer resistance protein—BCRP [20], and multidrug resistance-related proteins MRPs [21] as well as cytochrome P450 (e.g., CYP3A4, NADPH-CYP P450 reductase, etc.) [22] and Phase II detoxifying enzymes (such as UGT1A4), which help protect the brain from potentially harmful substances [19].

However, on the negative side, the same defense mechanisms apt to protect the brain from harmful substances also present and major hindering obstacle for drug delivery into the CNS. Recent studies have shown that approximately 98% of small-molecule and 100% of large-molecule drugs cannot cross the BBB [23, 24]. Thus the BBB is also a critical barrier hampering the treatment of major neurological disorders [25].

## Main text

### BBB dysfunction in neurological disorders

Historically dysfunctions of the BBB are associated with the onset and progression of different neurological disorders including Alzheimer’s disease [26], epilepsy [27], stroke [28, 29], multiple sclerosis [30], traumatic brain injury [31], amyotrophic lateral sclerosis [32] as well as schizophrenia [33]. Observed changes include alterations in BBB permeability [34, 35], caused by disruption and/or structural alteration of TJ proteins [36]. This process can be associated with the degradation of the basement membrane [37] and altered expression of efflux pumps leading to the extravasation of plasma proteins [38, 39]. The effect can lead to the infiltration of serum components and immune cells into the CNS parenchyma, loss of CNS homeostasis, and damage to the surrounding brain tissue. Similar to stroke, traumatic brain injuries (TBIs) cause both immediate and delayed dysfunction of the BBB, leading to inflammation [40] and the rapid activation of the coagulation cascade [41]. In short, TBI can promote post-traumatic intravascular coagulation followed by a significant reduction of blood flow in the pericontusional brain tissue, thus setting the stage for a condition closely resembling a post-ischemic injury.

It is noteworthy to mention that in the case of several diseases, it is still debatable whether the disease conditions are caused due to a disruption of the BBB or whether the disruption of the BBB is the result of the disease condition (e.g., epilepsy). Even though BBB leakage is observed preclinically in most circumstances, the degree of leakage has been found to vary from widespread leakage to localized small leaks in different brain regions. By contrast, BBB dysfunction has been established as a critical early event in relapsing–remitting inflammatory MS progression [42, 43]. In comparison, BBB breakdown and enhanced permeability precede and leads to infiltration of encephalitogenic T cells, monocytes, and likely B cells into the brain. Furthermore, therapeutic options to improve the BBB have proven to limit MS disease progression [44, 45], thus establishing a robust cause-effect link between loss of BBB viability and MS.

### Contradictory findings across animal models of neurological diseases and their impact on human studies

The widespread failures in clinical trials associated with neurological disorders have resulted in questions on whether the existing preclinical animal models are genuinely reflective of the human condition [46]. It is widely accepted that close interactions of pericyte and brain endothelial cells are necessary for the optimal function of BBB [47]. However, Mihajlica et al. recently demonstrated that pericyte deficient mice (Pdgfb^ret/ret^) produced similar values for K_in_ for Diazepam, oxycodone, and paliperidone compared to control mice. There are no changes in the transport mechanisms between the diseased and control conditions. There are also significant discrepancies in animal models of different neurodegenerative and cerebrovascular disorders. In the case of Alzheimer’s disease (AD), genetically engineered mice expressing the mutant genes for both APP and PSEN1 (here collectively termed APP/PS1 mice) yielded valuable insights into the mechanisms and consequences of amyloid deposition in the intact brain.

There are also reports where animal models of disease have shown no BBB disruptions in certain neurological disorders, whereas other studies have shown BBB disruptions in those specific disease models [48]. These conflicting findings simply raise more questions than answers. Recently, Nga-Bien Ly et al. reported that there is no difference in BBB integrity between wildtype control and humanized Alzheimer’s disease animal models [49], whereas other groups’ findings suggested otherwise [50, 51]. It should be noted that increased BBB permeability through gadolinium leakage was observed in the hippocampus of patients with mild cognitive impairment (MCI) and several grey and white matter regions in early Alzheimer’s disease (AD) patients. However, the degree of disruption may vary from patient to patient, since only 25% of patients with MCI and 45–78% of early AD patients were found to have brain microbleeds [52–54]. In this respect, AD animal models have also had limited success in predicting clinical outcomes. It is now being argued that these disease models are mostly reflective of the asymptomatic phase of the disease [55]. In the case of amyotrophic lateral sclerosis (ALS), transgenic mice having a G39A mutant form of human superoxide dismutase (SOD1) showed no differences in permeability across disease model and wild type control groups for both small molecule and large molecule markers [56]. Regarding Huntington’s disease (HD), current rodent models provide a poor representation of the disease course and outcomes [57, 58].

Discrepancies related to animal models of different neurological disorders have been discussed below in brief:

#### Alzheimer’s disease (AD)

Characterizing various forms of Mendelian dementia such as familial AD (FAD) mutations in the amyloid precursor protein (APP) or presenilin 1 (PSEN1) genes significantly improved our understanding of AD pathogenesis. Studies have also shown RAGE, and LRP balance plays a significant role in amyloid-beta transport into and out of the brain through the BBB [59]. A reduction in brain LRP levels may also play a role in amyloid-beta peptide accumulation [60]. Genetically engineered mice expressing the mutant genes for both APP and PSEN1 yielded essential insights into the mechanisms and consequences of amyloid deposition in the intact brain. However, to this day, the AD animal models have been unable to predict success in the clinic. For example, the Tg2576 model strain, which is based on a single FAD transgene, confirmed the relationship between amyloid pathology and impaired performance on cognitive tests in a preclinical experiment. Tg2576 mice have been improved or cured more than 300 times using different molecules. Yet none of these remarkable preclinical findings seem to transition to the clinical phase. It is now being argued that these disease models only helped address the early/asymptomatic stage of the disease [55].

#### Huntington’s disease

Huntington’s disease (HD) is an autosomal dominant inherited invariably fatal disorder. It results in mutated Huntington proteins that aggregate in different neurons. Over the course of the disease, there is significant neuronal cell loss, and patients show typical phenotypic hallmarks such as cognitive deficits, personality disorder, and hyperkinetic movements [61]. Studies have found that the BBB is disrupted in an animal model of HD (R6/2). Corresponding morphological changes were also observed in post-mortem tissues from human patients [62, 63]. Similar findings, however, are yet to be seen in live human subjects. To date, over 25 transgenic rodent models of HD have been used, yet none are able to effectively reproduce the neurodegeneration features and disease progression patterns that has been clinically observed [57, 58].

#### Parkinson’s disease

Parkinson’s disease (PD) is a movement, and cognitive disorder with many pathologic hallmarks, including the formation of proteinaceous inclusions inside neurons, called Lewy Bodies and loss of dopaminergic neurons in the Substantia Nigra pars compacta [64]. PD has been found to be associated with multiple mutations. However, no single genetic anomaly was proven to be causing PD. The disease is thought to be linked to a range of polygenetic and environmental factors. 1-methyl-4-phenyl-1,2,3,6-tetrahydropyridine (MPTP) is a lipophilic compound and crosses the BBB easily and was found to induce symptoms and pathology of PD in animal models [65]. However, the injury-induced models mimic nigrostriatal dopamine deficiency but do not recapitulate the slow, progressive, and degenerative nature of the disease in humans. Whereas in clinical trials, the interventions are usually administered over a prolonged period of time, putative neuroprotective agents were being delivered at similar doses and schedules as an acute Parkinson’s disease-like lesion, which was induced in the typical underlying animal studies [66].

#### Stroke

Stroke incidence increases with age, and patients commonly have other comorbidities that might increase their stroke risk, which complicates the clinical progression and affect the functional outcome. Up to 75% of acute stroke patients have hypertension, and 68% have hyperglycemia as comorbidities [67]. On the contrary, only 10% of focal ischemia studies used animals having hypertension, and less than 1% used animals had induced diabetes. The preclinical studies also are mostly performed in young animals. This latter is because achieving appropriate infarct volumes in older animals as well as animals that have additional disease conditions using conventional techniques (such as middle cerebral artery occlusion) is quite tricky and prone to high variabilities. The animals used in different stroke models are usually young and mostly males. More than 95% of the studies were done on rodents. Larger animals that would be biologically closer to humans are rarely used [68]. Most preclinical studies also fail to acknowledge the delay between the identification of symptoms of stroke and the start of treatment, which is typically a couple of hours for most stroke patients. Also concerning the post-stroke assessment of BBB integrity as well as in other conditions where the integrity of the barrier might be compromised, gadolinium-based studies of microvascular integrity performed have shown limited sensitivity to detect low levels of extravasations across the BBB [69]. The facts discussed above highlight the urgent need to utilize technology that would better represent the disease conditions in humans.

### Importance of permeability studies in advanced BBB-on-chip models

In recent years, significant steps had been taken toward developing physiological BBB-on-chip models. These recently described advanced microfluidic models provided 3D structure, cell–cell interaction, and the exposure to shear stress that resulted in better barrier function compared to conventional transwell models [70–73]. Moreover, they have characterized the dynamic permeability of drugs/markers, which make them more like in vivo permeability studies compared to the Transwell system [70, 72, 74]. However, there are still challenges ahead for developing in vitro model of BBB due to different designs of models and quantitative protocols. We can see the difference in the design and size of microchannels, shear stress, cell types, and permeability measurements in Table 1. Based on the versatility of BBB-on-chip technology, the comparison of these models would be complicated. The BBB maintains a unique homeostatic environment within the CNS and plays a critical role in mass transfer between the circulatory system and brain tissue. Therefore, permeability measurements in BBB-on-chip models and data comparison with corresponding in vivo studies are usually performed as a means of understanding whether the model can surrogate for *the* in vivo study and whether an appropriate in vitro to in vivo correlation can be established. In the following part, we will provide more information regarding the process of measuring permeability in BBB-on-chips.

**Table 1:** Overview and characteristics of current BBB-on-chips models

Date published	Microvessel/endothelial compartment size	Material and membrane	Biological coating	Endothelial cell type	Additional cells in co-culture (type)	Barrier permeability tracer	Permeability measurement (cm/s)	TEER (Ω cm^2^)	Shear stress (dyne/cm^2^)	Ref#
2019	Microfluidic device (200 μM width, 100 μM height)	PDMS	Human fibronectin (300 μg/ml)	Immortalized human cerebrovascular endothelial cells (hcmec\D3)	Astrocytes cultured in basolateral compartment	10 kDa and 70 kDa FITC dextran	10 kDa FITC dextran: 15 × 10^−6^ cm/s 70 kDa FITC dextran: 3.7 × 10^−6^ cm/s	NA	2.73	[143]
2019	150 μM	PDMS	Collagen type IV and Fibronectin	BMECs derived from human iPSCs	N/A	Lucifer yellow, Alexa 647 conjugated 10 kDa dextran	Lucifer yellow: 5–6 × 10^−7^ Alexa 647 conjugated 10 kDa dextran: below detection limit	2000–4000	1	[144]
2019	(Two channels) 2 cm long and 1 mm wide; top and bottom channels were 1 and 0.2 mm high	PDMS), 2-channel, separated from a parallel vascular microchannel by a porous (2 μm diameter), polyethylene terephthalate (PET) membrane	Collagen type IV and fibronectin	iPS-BMVECs	Primary human pericytes and astrocytes	Fluorescent labeled of dextran tracers (3, 10, or 70 kDa)	8.9, 1.1, and 0.24 × 10^−8^ cm/s for 3,10, and 70 kDa	24,000^a^	6	**[**70**]**
2019	150 μm	Collagen type 1 rat (hydrogel)	Collagen type IV and fibronectin	iPS-BMVECs	NA	Lucifer yellow, Rhodamine 123 (R123), and 10 kDa dextran	LY = 2.84 ± 0.41 × 10^−7^ cm/s R123 = 1.32 ± 0.16 × 10^−7^ cm/s The permeability of 10 kDa dextran in dhBMEC microvessels was below the detection limit	NA	4	[71]
2019	Cylindrical template rod (150 μm)	Collagen type 1 rat hydrogel	Collagen type IV and fibronectin	Human iPSC-ECs	Human iPSC-pericyte	Lucifer yellow, and 10 kDa dextran	LY = 4 × 10^−7^ cm/s The permeability of 10 kDa dextran was below the detection limit	NA	4	[145]
2018	Self orgnized less than 100 μm	Fibrin gel	Fibronectin	Human iPSC-ECs	Primary human pericytes and astrocytes	40 and 10 kDa FTIC-dextrans	Of 8.9 × 10^−8^ cm/s and 2.2 × 10^−7^ cm/s for 40 kDa and 10 kDa FTICdextran,	NA	NA	[146]
2017	Microfluidic chip with fluidic channels (190 μM height and 920 μM width)	PDMS, hydrogel channel is 580 μM wide while the fluidic channel is 920 μM wide. Endothelial cells seeded in the fluid channels. Astrocyte and Neurons mixed in collagen-1 and perfused in the hydrogel channels	Poly-d-Lysine	HUVEC or hcmec\D3	Primary cortical neuron from rat, primary astrocytes from rat	10 kDa Oregon green 488 dextran, 70 kDa Texas red dextran	10 kDa Oregon green 488 dextran in HUVEC: 7 × 10^−5^ cm/s 70 kDa Texas Red Dextran in HUVEC: 5 × 10^−5^ cm/s 10 kDa Oregon green 488 dextran in hcmec\D3: 1 × 10^−5^ cm/s 70 kDa Texas Red Dextran in hcmec\D3: 0.25 × 10^−5^ cm/s	NA	NA	[73]
2017	Microfluidic device (width 2 mm and height of 0.2, 0.6 and 1 mm)	PDMS with polyester membrane (0.4 μM)	Fibronectin solution	Immortalized mouse endothelial cells (Bend3)	Immortalized mouse pericytes adhering to polyester membrane on 2nd channel and mouse astrocyte type I clone on the bottom of 2nd channel	10 kDa Oregon green 488 dextran 70 kDa Texas red dextran	Monoculture: HUVEC: 7 × 10^−5^ cm/s (10 kDa), 5 × 10^−5^ cm/s (70 kDa) hcmec\D3	Single culture: ~ 134 Bi culture: ~ 260 Triculture: ~ 310	NA	[147]
2017	(300 μm * 160μm)	Polycarbonate insert with 04 μm pore size	Collagen type IV and fibronectin	Human iPSC-ECs	Rat primary astrocytes	4, 20 and 70 KDa FITC-dextrans	4 kDa = 10^−7^ and 20 and 70 was close to10^−8^ cm/s	2000–4000	0.25	[72]
2017	180 μm cylindrical rod	Collagen type I hydrogel in PDMS	Collagen	(hCMEC/D3)	Human astrocytes	4 kDa FITC-dextran	0.8 × 10^−6^ cm/s	200-1000	0.5	[148]
2016	PDMS with collagen hydrogel inside (1 mm * 1 mm)	Collagen type 1 rat	NA	Primary human endothelial cells	Primary human pericytes and astrocytes	3 kDa fluorescent dextran	2–4 × 10^−6^ cm/s	NA	1	[149]
2016	Two rectangular PDMS channels with 3.7 cm × 0.2 cm × 0.2 cm size	Polycarbonate membrane with a thickness of 23 μm and a pore size 0.45 μm	Rat tail collagen	hCMEC/D3	NA	Sodium fluorescein (376 Da), 4 kDa FITC-dextran, f Evans blue-labeled albumin (67 kDa)	Sodium fluorescein = 1.57 × 10^−6^cm/s 4 kDa Dextran = 1.32 × 10^−6^ Evans blue = 0.15 × 10^−6^cm/s	28	0.15	[150]
2016	Two rectangular PDMS channels with 3.7 cm × 0.2 cm × 0.2 cm size	Polycarbonate membrane with a thickness of 23 μm and a pore size 0.45 μm	Rat tail collagen	Primary rat brain endothelial cells	Rat primary pericytes and astroglia	Sodium fluorescein (376 Da), 4 kDa FITC-dextran, f Evans blue-labeled albumin (67 kDa)	Sodium fluorescein = 1.15 × 10^−6^cm/s 4 kDa Dextran = 0.20 × 10^−6^ Evans blue = 0.04 × 10^−6^cm/s	114.2	0.15	[150]
2015	Microfluidic chip with vascular channel having 100 μM height	PDMS, 0.2 μM Polycarbonate membrane	Laminin	Primary hBMVEC	Primary astrocytes, and pericytes plus neurons differentiated from hiPSCs	10 kDa and 70 kDa FITC Dextran	Not measured. Only absolute fluorescence measured	Custom built impedance measurement (~ 12,000^a^)	NA	[96]
2015	3D printed vessels having 235 and 260 μM diameters	3D co-printing Vero White Plus-FullCure 835 resin as main framework and poly-vinyl alcohol (PVA) as a dissolvable support material	Fibronectin	Bend3 cells	N/A	40 kDa FITC dextran	2.27 × 10^−7^ cm/s	NA	NA	[151]
2015	Rectangular PDMS channel (6.3 mm × 100 μm)	PDMS	Fibronectin	Neonatal rat brain capillary endothelial cells	Primary cultures of neonatal rat astrocytes	10 and 70 kDa fluorescent dextran	2.9 ± 1.0 × 10^−6^	150-180	3.8 × 10^−3^	[152]
2015	Rectangular PDMS channel (1 mm × 150 μm)	PDMS	Collagen IV–fibronectin	bEnd3	Immortalized mouse astrocyte C8D1A	70 KDa dextran	5.9 × 10^−7^ cm/s	NA	5	[153]
2013	Rectangular PDMS channel (200 μm × 100 μm)	PDMS	Fibronectin	Rat brain EC (RBE4 cells)	N/A	3 and 5 KD FITC-dextran	Relative comparison only	NA	NA	[154]
2013	PDMS channel (100 μM × 500 μM)	PDMS	Fibronectin	Rat brain EC (RBE4 cells)	E18 rat cortical cells	3 kDa Alexa fluor-dextran	Relative comparison only	NA	NA	[155]
2012	Rectangular PDMS channel with a length of 1 cm, a width of 500 μm and a depth of 100 μm	Polycarbonate membrane with a thickness of 10 μm and a pore size of 0.4 μm	Rat collagen type I	(hCMEC/D3)	N/A	N/A	N/A	120	5.8	[156]
2011	11 hollow polypropylene fibers (600 ± 90 μM) inside a sealed chamber (the extraluminal space) accessible by ports	Polypropylene with transcapillary pores (2 to 4 μM)	Fibronectin	Normal adult human brain microvascular endothelial cells	Human adult astrocytes	Sucrose, phenytoin and diazepam	Sucrose: 3.16 × 10^−6^, phenytoin: 6.75 × 10^−5^, diazepam: 6.88 × 10^−3^ cm/s	~ 500 on the 21st day	4	[157]
2002	11 hollow polypropylene fibers (600 ± 90 μM) inside a sealed chamber (the extraluminal space) accessible by ports	Polypropylene hollow fiber apparatus with 12 μM pores	Fibronectin	Bovine aortic endothelial cells (BAEC)	Rat glial cell tumor line	N/A	N/A	500	NA	[90]

“N.A.” shows that the specified aspect has not been measured or reported

^a^Indicates the TEER value reported were not normalized based on the surface area of the microfluidic chip

The permeability results of recent in vitro models have been reported in Table 1. Defining quantitative standards for barrier function is difficult for in vitro models since all quantitative data comes from animal models. The permeability values are described as the permeability coefficients of an analyte (cm/s) for passive transport. These values can be compared with in vivo values due to their independence to the analyte concentration, flow rate, and channel size. The values were obtained by injecting different molecular weight markers having fluorescent labels into the vascular channel and calculating mass conservation based on the number of fluorescent molecules outside the vessels. Equation (1) can be used to obtain the permeability of different fluorescent molecules for advanced 3D models.

$$p = \frac{1}{\Delta I}\frac{V}{{A\left( {surface} \right)}}\frac{dI}{dt}$$

here, V is the tissue volume, A (surface) is the surface area of all vessels in the selected ROI (region of interest). ΔI is the maximum fluorescence intensity of the vascular channel, and (dI/dt) is the rate of increase in fluorescence intensity as solute exits into the tissue compartment. Afterward, the permeability coefficient of the endothelial barrier can be calculated from the measured permeability coefficient (P total) and the permeability coefficient measured in a device without endothelium P (blank) as follows.

$$Pendo = \frac{1}{Ptotal} - \frac{1}{Pblank}$$

Therefore, the obtained endothelial permeability coefficient can be compared with other permeability coefficients of the same analyte in vivo or other platforms, including the Transwell system.

Recently published studies related to the use of induced pluripotent stem cells (iPSC)-in microfluidic BBB platforms have found that these cells, under suitable culture conditions, temporarily developed into a BBB phenotype with permeability values close to that measured in vivo. In one study of tissue-engineered BBB microvessels incorporating iPSC-derived human BMECs, the permeability of Lucifer yellow was reported to be 2–3 × 10^−7^ cm/s [71] which was close to values reported in a rat model earlier [75]. In another study of a 3D self-organized microvascular model of the human BBB with endothelial cells, pericytes and astrocytes, the permeability 40 kDa FITC-dextran under mono-, co-, and tri-culture conditions were 6.6, 2.5, and 0.89 × 10^−7^ cm/s, respectively. These results show that the presence of co and tri-culture reduces the permeability of analyte. Similar results were obtained for 10 kDa FITC-dextran: 12, 4.8, and 2.2 × 10^−7^ cm/s, respectively. These values where comparable to those measured in vivo in rat cerebral microcirculation (3.1 ± 1.3 × 10^−7^ cm/s for a 10 kDa FITC-dextran) [76], (1.37 ± 0.26 × 10^−7^ cm/s for a 40 kDa FITC-dextran) [77]. Moreover, the permeability study of different molecular weight fluorescent-labeled dextran tracers in recent study of iPSC-BMECs in 3D microfluidic chip with presence of primary astrocyte and pericytes was deficient and the permeability values inversely correlated with the size of the tracer (average Papp = 8.9, 1.1, and 0.24 × 10^−8^ cm/s for 3, 10, and 70 kDa dextrans, respectively) [70].

The above studies indicate that the models are moving in the right direction for proper in vitro in vivo correlations (IVIVC), something that was not feasible utilizing the transwell systems. Based on the translational challenges as well as ethical concerns and economic implications of small and large animal testing, it has become very crucial to develop a humanized BBB model encompassing the cell typologies represented in the NVU. These humanized models could help us properly understand changes in the NVU in disease conditions. There are also some reported devices trying to replicate the neurovascular unit and disease conditions [78–80]. However, we are still not there yet where we can produce diseased BBB conditions that will contain iPSC-derived endothelial cells, astrocytes, pericytes, and neurons in advanced in vitro models. Hopefully, with further improvements in techniques, we will be able to get there soon. Noteworthy is the fact that most research groups are still using large molecule dextrans for measuring passive permeability, which technically should not be able to get into the BBB in vivo at all in naïve conditions. Only Searson’s group recently reported a study utilizing Lucifer yellow and 10 kDa Dextran, where the 10 kDa Dextran did not permeate the barrier at all. There are some reports where the models have been used in conditions representing brain inflammation [70]. However, we still must improve these models further to effectively produce disease models that would perfectly represent in vivo conditions.

#### Importance of developing in vitro models of human BBB

To date, various in vitro BBB models have been developed and characterized in terms of barrier tightness, expression of BBB specific proteins, and usefulness for physiological and pharmacological studies [3, 81]. Traditionally the transwell systems utilizing immortalized endothelial cells were not producing tight barriers and had low TEER values. Higher molecular weight markers such as FITC dextran were used regularly for such systems since the lower molecular weight markers would pass through relatively quickly. As discussed earlier, animal models do not always recapitulate the human BBB physiology or a disease condition (including pathological characteristics such as onset, progression, etc.). Unavoidable interspecies differences are likely to play a major role. For example, works done by Terasaki et al., using QTAP technology clearly demonstrated that the mouse BBB is different from the human BBB [82]. Furthermore, intraspecies variabilities can equally impact data reproducibility thus affective the translational relevance of the results [83]. Tentative in vitro models based on immortalized human brain endothelial cells have also been proposed in static or dynamic conditions [40, 84]: these models recapitulate at least some of the expected characteristics of the BBB, like high level expression of BBB-expressed receptors and transporters. Therefore, “humanizing” the in vitro BBB platforms using human-derived cells, may circumvent the translational limitations of current models and provide a complementary tool to support existing in vivo approaches. However, one of the major limiting factors to reproduce a “humanized” in vitro model to study neurological disease or disease state includes the availability of human brain tissues for cell isolation. This is quite difficult to procure and mostly originate from either post-mortem specimen (including fetal tissue) or patient-derived surgical resections. This latter, although may not represent the actual conditions in the native state.

Although most in vitro BBB models currently available are based on primary cultures of cerebral endothelial cells, endothelial progenitor cells (EPCs) might be other alternatives that present a specific phenotype of EC [85]. In these systems, cord blood-derived hematopoietic stem cells are used to generate a reproducible and stable human BBB model where the cells are initially differentiated into endothelial cells (ECs). Following the differentiation process, these ECs are then prompt to develop BBB properties by co-culture with pericytes [86].

These models were found to form much tighter barriers that can mimic human BBB integrity. However, there are still challenges ahead for developing the in vitro model of BBB due to different designs of models and quantitative protocols. Key features of recent microfluidic and 3D printed models of BBB has been summarized in Table 1.

**Fig. 2:**
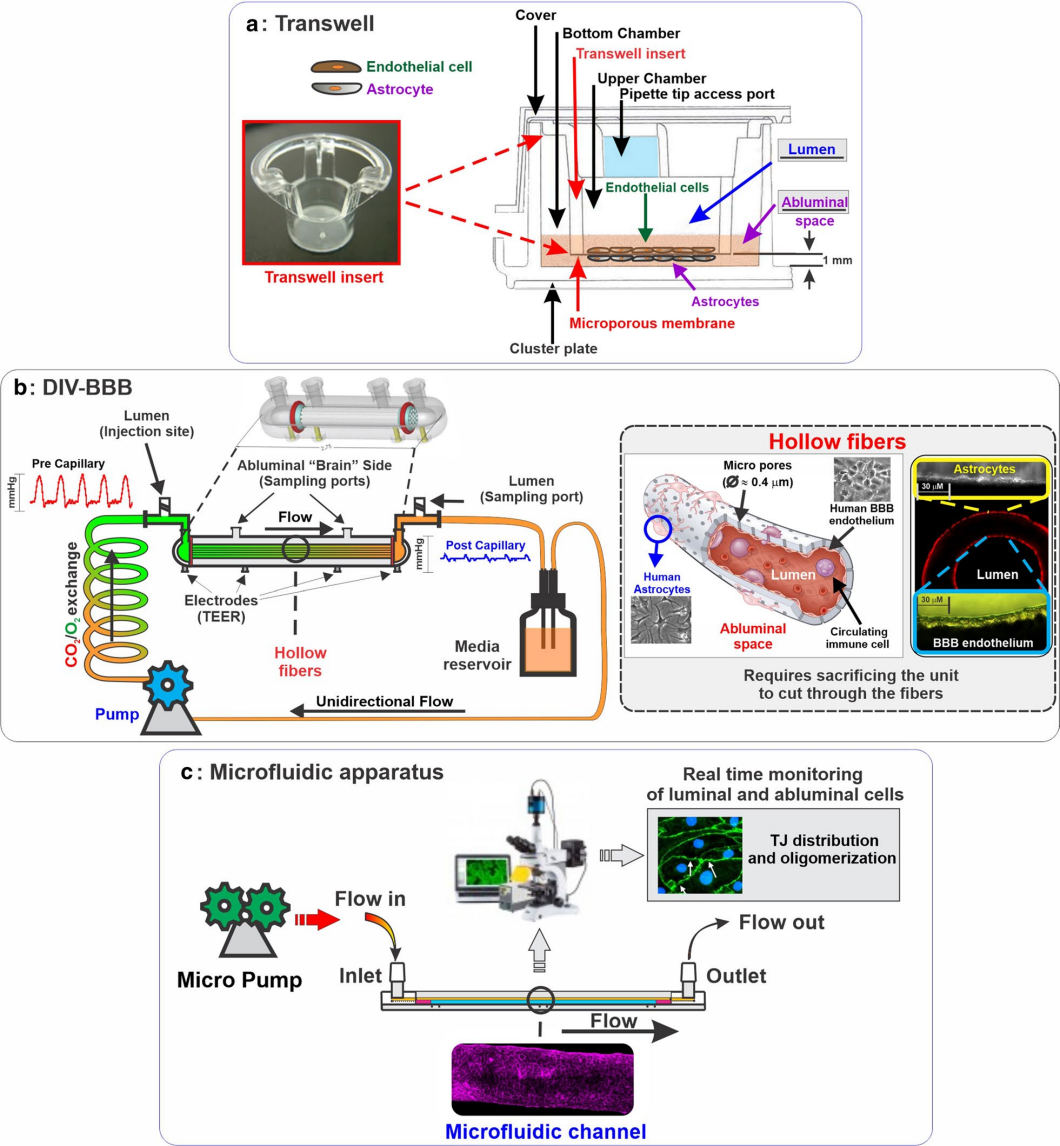
Side by side schematic view of various in vitro BBB platforms. **a** The Transwell apparatus which consists of a vertical side by side diffusion system across a semipermeable microporous membrane. The membrane allows for free passage of nutrients and diffusible factors between the luminal and abluminal compartments. Depending on the membrane’s pore size, cell extravasation across the compartments can be enabled. **b** Dynamic in vitro BBB model (DIV-BBB). This platform relies on the use of hollow fibers to simulate the architecture of a blood vessel. The hollow fiber can be pre-coated with specific coating factor to enable the adhesion of endothelial cells (generally on the luminal surface of the fiber) and astrocytes or other NVU cell types on the abluminal surface in juxtaposition to the endothelium. A pulsatile pump generates the medium flow across the system, mimicking the blood flow traveling inside the blood vessel. The bundle of hollow fibers is suspended inside a sealed chamber. The artificial capillaries are in continuity with a medium source through a flow path consisting of gas-permeable silicone tubing. Ports positioned on either side of the module allow access to the luminal and abluminal compartments. The system allows generating rheological conditions like those observed in vivo. It also allows for the perfusion and circulation of immune cells as required. **c** Schematic illustrations of a typical microfluidic platform. The system recapitulates the characteristic of a DIV-BBB but to a much smaller scale. Most of these platforms also enable visual assessment of the cell environment through visual microscopy (including fluorescent, and confocal) to assess cell morphology, distribution, cell contact, etc. Some of the limitations inherent to microfluidic systems include the very small sampling size (for qualitative quantitate assessments) and lack of availability to other researchers with very few exceptions

Although cell culture-based in vitro models are useful tools to study the regulatory mechanisms modulating the physiology and function of the BBB as well as assess the passage and transport mechanisms of putative brain-penetrating drugs, reproducing the BBB properties in its entirety remains a significant and still unresolved challenge [87]. Different approaches have been used to mimic the BBB in vitro, and this includes static and dynamic (flow-capable) platforms, as well as the use of different cell types such as primary cells, immortalized cell lines, and, more recently, stem cells. In addition to using different cell types, cell cultures for BBB modeling have grown in structural complexity ranging from basic monocultures to multiple culture systems such as co-culture and tri-culture settings [17, 19]. The transwell system is one of the most commonly used tools as a BBB in vitro model. Although being very user-friendly and relatively easy to setup (Transwells also offer moderate scalability and high throughput screening—HTS—capabilities [40, 41]), there are substantial limitations inherent to the use of these platforms that need to consider. These include the two-dimensional structure, absence of enabling endothelial exposure to shear stress, and “edge effects” where areas of the transwell walls surrounding the membrane are intrinsically very permeable [41, 44, 45] (see Fig. 2a).

Further technological advances were then introduced to enable exposure of the BBB endothelium to physiological shear stress (SS), whereas SS modulates endothelial morphology [88], but also their function and physiological responses paving the way toward the development of the so-called “Dynamic Models.” Artificial hollow fiber constructs made of thermoplastic polymers such as polysulfone, polypropylene, etc., were initially used for the construction of brain microvessels and other CNS vascular beds [89] where EC-glia co-culture could be arranged to mimic the spatial and topographical distribution of these cells in situ resembling the anatomy of brain microvessels [90]. Under these controlled hemodynamic conditions combined with exposure to glial cells, ECs acquire more stringent BBB properties than those observed static platforms, including high TEER [40], cell polarization, and expression of specialized transporters [90] and efflux systems [40, 91] (see Fig. 2b).

However, the enthusiasm for the add on advantages brought by this dynamic in vitro-BBB (DIV-BBB) platform is hampered by additional drawbacks inherent to its design and construction. Since the model relies on the use of capillary-like tubes structures surrounded by a larger enclosure, no practical way exists to monitor the cells cultured on or within the artificial microvessels. The relatively large diameter of the capillaries compared to proper brain microvessels was more representative of larger vascular beds like distal pre- and post-capillary segments. The DIV-BBB platform required a relatively large volume of reagents and high quantities of cells (on the magnitude of > 10^6^) for culture initiation, thus affecting the cost, and the model per se does not have high throughput screening (HTS) capabilities. Furthermore, the system setup was quite complex, requiring a significant amount of time, resources, and technical skills than conventional platforms (e.g., Transwells) [91, 92]. Moreover, the most dynamic and realistic in vitro BBB model, microfluidic devices, requires specific equipment and technical skills mostly confined to the lab environment that developed the platform. These constraining factors, unfortunately, limit the adoption and further development of these models for basic and translational research [87] (see also Fig. 2c).

In-silico models have also been developed to estimating structure–activity relationships for the BBB permeation of drug compounds based on their physicochemical properties [93–95]. Although these models are inexpensive, less time consuming, and high throughput screening methods for novel compounds in the drug discovery process, results obtained using computer simulation must be verified by in vivo experiments [96].

### Advances in *BBB* in vitro modeling

#### Organoids

An organoid is an in vitro organotypic preparation consisting of various cells grown together under appropriate conditions to generate a miniature artificial version of an organ of interest, including the brain [97]. BBB organoids consist of human primary brain endothelial cells, astrocytes, and pericytes [98] assembling under low-adherence conditions into a multicellular structure resembling the blood–brain barrier [99]. One of the important characteristics of this model is that within the organoid, each of the cell types is directly in contact with one another, which plays a pivotal role in maintaining BBB integrity as well as function [100]. It has also been demonstrated from various studies that organoid shows better characteristics of BBB, which includes enhanced tight junctions, adherens junctions, and efflux pump expression compared to more traditional static culture systems. Additionally, lack of paracellular permeability, high drug efflux, and receptor-mediated transcytosis infer a realistic barrier function to the organoids [98]. Furthermore, using an organoid in vitro BBB model consisting of ECs, astrocytes, and pericytes, a recent study reported high expression of TJs proteins, VEGF dependent permeability, receptor-mediated transcytosis of angiopep-2, as well as activity of efflux pumps.

On the other hand, the transwell model exhibited a lower level of BBB regulatory proteins, and the differentiation between the transportation of angiopep-2 and a control peptide was not possible. In this experiment, two different detection approaches, namely confocal fluorescence microscopy and MALDI mass spectrometry imaging, were used as a screening tool [98]. More recently, Pham et al. have developed a human vascularized cerebral organoid model utilizing a patient’s iPSCs to study the BBB under normal and pathological conditions in health and disease [101].

To sum up, organoid BBB models seem to offer several advantages over other conventional in vitro BBB platforms, including but not limited to HTS due to ease of culture, simplicity, low requirement of reagents, and miniature size [87]. The throughput of this model can be further improved through integration with automated microscopy and robotics-assisted mass spectrometry technologies. Cost-effectiveness and reproducibility make this model more acceptable and attractive to implement in research as well. Considering the advantages offered by BBB organoids-based in vitro models, it is evident that the technology provides an efficient method for studying drug transport through the BBB and practical support for the development of brain-targeting drugs for the treatment of CNS diseases [98].

Nevertheless, organoids have some limitations, as well. The issues of inter-samples variability and high processing time are not negligible. One of the critical drawbacks of organoids is the absence of essential types of cells, including glia, microglia, oligodendrocytes, vasculature, etc. Moreover, the timescale of human development, lack of microglia, regional inputs, and myelination may hinder neurons maturation, thus limiting its utilities for specific disease models [87, 102]. Furthermore, methods of barrier function analyses from two dimensional cultures will have to be adjusted to the analysis of 3D organoid structures. Few attempts have been made to develop this methodology to assess the permeability of a compound in a 3D organoid and so far, these methods have been primarily developed for measurements of the barrier permeability in intestinal organoids [103].

#### Human cortex spheroid in vitro BBB models

Focusing on the drawbacks of conventional organoid in vitro BBB model, recently, the development of a 3D spheroid model of BBB has been reported. This model more closely mimics the human brain tissue since it is comprised of six cell types found within the brain cortex. These cell types include human brain microvascular endothelial cells (HBMEC), human pericytes (HBVP), human astrocytes (HA), human microglia (HM), human oligodendrocytes (HO) and human neurons (HN), with endothelial cells enclosing the brain parenchymal cells.

Induced pluripotent stem cells (iPSC) were used to derive the cells which could help narrow the gap in achieving an ideal in vitro BBB model for clinical applications in studies aimed to understand neurological diseases. Interaction between BBB and adjacent brain cells provides a platform to evaluate the ability of a novel drug to cross the BBB and its effect on microglia, oligodendrocytes as well as neurons, which is crucial for studying neurodegenerative conditions including amyotrophic lateral sclerosis, multiple sclerosis, stroke, Alzheimer’s disease. High cell viability was found to be maintained up to 21 days in the spheroid model containing six cell types, which is useful in evaluating long term effects of drug toxicity [104]. Expression of P-gp and GLUT-1 proteins were also identified as these proteins have a pivotal role in expelling unwanted chemical from the brain tissue and transport glucose into the brain tissue respectively and abnormalities in these proteins lead to different diseases [104–107]. Expression of tight junctions, adherens junctions, and proteins associated with adherens junction was also identified to avert the free paracellular diffusion of substances into the brain parenchyma. Additionally, it was also found that tight and adherens junction protein localization was disrupted by hypoxia, thus supporting the usefulness of this model to study ischemia. BBB selectivity was also evaluated by measuring the effect of mercury ions in brain parenchyma by assessing cell viability [104].

Considering the characteristics and features of this model, we can state that, human cortex spheroid in vitro BBB model may provide some additional advantages over conventional organoids and can be a suitable platform for studies related to drug discovery, disease modeling, and neurotoxicity. However, further structural consideration to identify the production and proper deposition of extracellular matrix proteins of BBB and analysis of effects on individual cell types to evaluate cell-specific function is needed, which will make this model more acceptable to study different neurological diseases [104]. A more comprehensive description of CNS-related organoid models, including similar techniques, protocols, use, and limitations, has been recently published by Pacitti et al. elsewhere [101].

#### 3D ECM-based BBB models

One of the significant hurdles in the field of in vitro modeling that current technologies have been trying to overcome is that of providing a quasi-physiological microenvironment to promote the BBB development of realistic physiological properties and responses to endogenous as well as exogenous stimuli [108, 109]. 3D in vitro tissue models are currently available for a variety of organs and tissues (including muscle, bone, liver, and cardiac tissues) [110]. The use of this technology is now making a modest appearance in cerebrovascular and BBB research, as well.

**Fig. 3:**
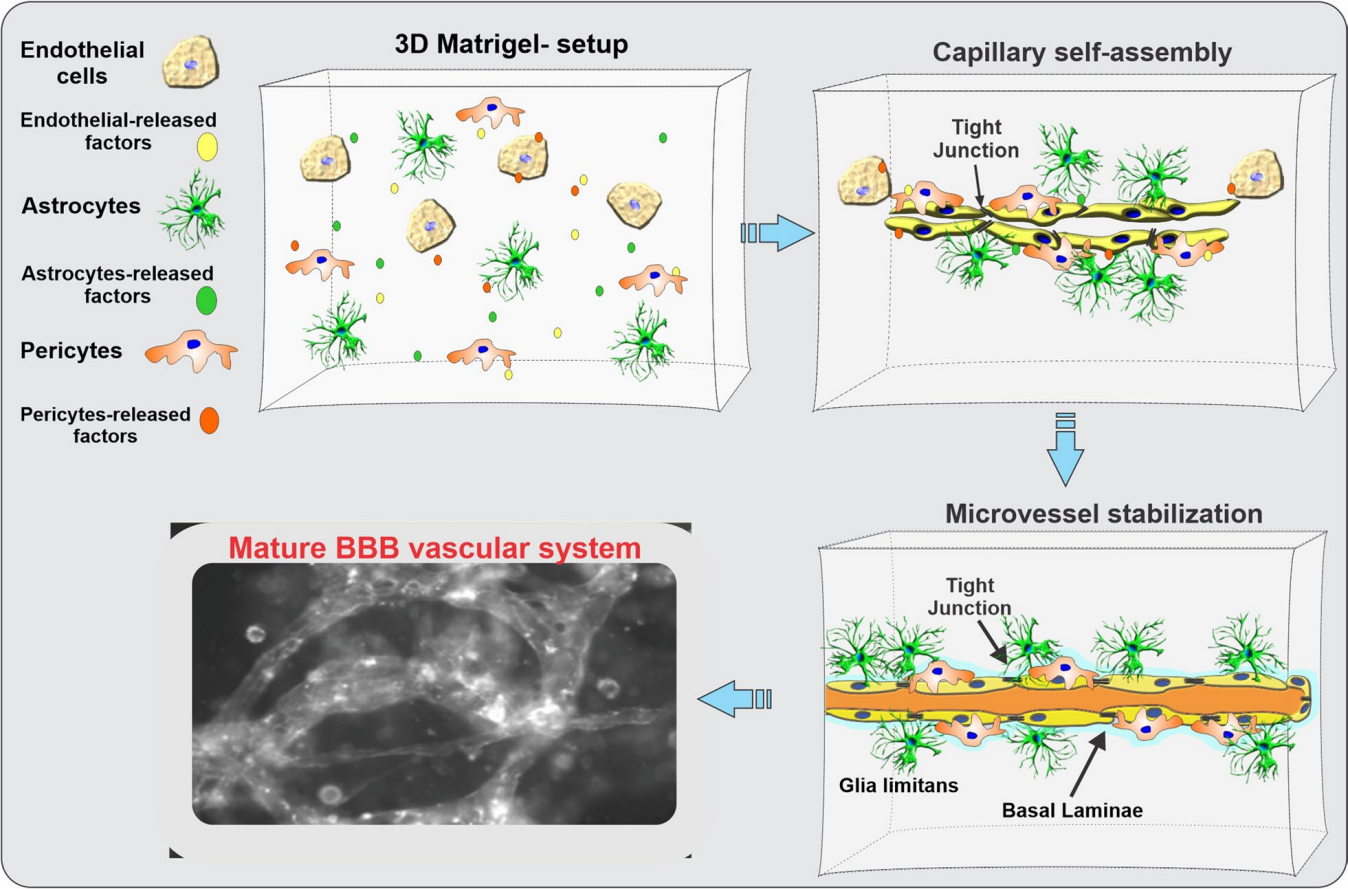
Schematic representation of a 3D ECM-based in vitro BBB model. This platform enables culturing multiple cell types (related to a specific organ system) at once. The formation of natural gradients of biological factors (either introduced into the matrix and/or naturally produced by the cells in culture) promotes a host of physiological responses (including cell migration, interaction, and differentiation) culminating with the self-assembly of microvascular processes and the formation of a network of capillary-like structures in vitro

In the specific case, the brain microcapillaries are grown on self-polymerizing extracellular matrix protein (ECM) scaffolds where the BBB cellular components can develop close interactions with one another while being exposed to trophic factors along quasi-physiological biochemical gradients (see Fig. 3). High-resolution confocal microscopy and/or other 3D imaging techniques such as multiphoton microscopy and optical coherence tomography can be used to monitor the dynamic changes of cells cultured in the 3D ECM microenvironments.

However, the adoption of this technology in BBB research is still limited due to the complexity of developing an in vivo-like matrix architecture, where the omission of even minor ECM constituents can potentially alter the matrix property and thus architectural assembly processes of the microvasculature. These platforms are currently confined to basic research with relatively low translational/pharmaceutical appeal due to several factors including high complexity, lack of HTS capabilities, consistent reproducibility, etc.

#### Microfluidics via 3D printing

In recent years, in the field of biomedical research, microfluidics has emerged as a promising alternative due to its high throughput, automation capabilities, and low fabrication and operation costs [111]. However, current microfluidic devices have relied on multi-step lithographic processes, which are time-consuming and complicated. In order to solve this critical issue, currently, 3D-printing (additive manufacturing) is becoming an alternative approach to microfluidic fabrication with complex architectures, avoiding multi-step processing with a wide range of materials [112–114]. In fact, 3D printing, as digital fabrication technology, is a process of adding elements to fabricate objects from 3D model data, layer by layer, enabling precise construction of complex objects directly from a computer-aided design (CAD) software [115]. Indeed, 3D printed microfluidic technology provides researchers with several advantages over traditional fabrication techniques including the ability to build channels with unprecedented shape and complexity that are uniform and reproducible at minimal operating cost and time (reduced from weeks to a few hours), product complexity, reduction of user error, precisely controlled size, interconnectivity and geometry, flexibility and throughput [112, 116–118].

A similar procedure is used in most 3D printing processes for manufacturing solid structures from digital designs. Briefly, the intended product is digitally rendered in 3D with computer-aided design (CAD) software, and then 3D designs are converted to the stereolithography file format (STL), describing the external surface of a 3D model [116]. The data is then further sliced into a build file of 2D layers and sent to the 3D printing machine [115]. Raw materials such as thermoplastic polymers (including acrylonitrile butadiene styrene—ABS, polylactic acid—PLA, polyamide—PA, and polycarbonate PC), natural polymers, and biocompatible synthetic polymers [114, 116, 119–123] are processed into filaments or granules. Then binder solutions are added and solidified automatically in a layer-by-layer manner to produce the desired product. After printing, products may require polishing, drying, sintering, or other post-processing steps. Unprinted materials will also be harvested and recycled for continued use in the printing process [124]. Various printing techniques have been utilized for microfluidic applications. The leading 3D set up processes for microfluidic systems are 3D Printed Transfer Molding (PTM), Fused Deposition Modeling (FDM), Stereolithography (SLA), Direct Ink Writing, and Selective Laser Sintering (SLS) [124–126].

Recently, several new techniques have been developed for 3D printing, including Poly jet, digital light processing (DLP), liquid deposition modeling (LDM), and fiber encapsulation additive manufacturing (FEAM) [127–130]. Although these methods have more material selections or less processing time, only a few studies have been conducted to assess the viability of these techniques to manufacture microfluidic systems, due to their high cost and complexity compared to traditional 3D printing methods. A more comprehensive description of 3D printing techniques, protocols, use, and limitations has also been recently published by Sivandzade et al. elsewhere [22].

Despite the potential advantages provided by 3D printing fabrication processes in BBB modeling, the technology is not yet mature, with several limitations that still hinder its widespread adoption. For example, the lack of high-throughput 3D-bioprinted tissue models for research makes this technology not yet suitable for drug discovery and toxicology studies. The complexity of the tissue to be reproduced increases exponentially the complexity of the technical challenges that need to be overcome. These include conjugating multiple elements such as fabrication materials, cell types, cell distribution as well as loading of the necessary biological factors to maintain cell viability, and construction of the tissue scaffold itself. However, to advance this up and coming technology, any further will require the integration of multiple fields of research, including engineering, biomaterials science, cell biology, physics, and medicine.

An additional variable to consider (and a potential challenge) for the development of viable and clinically relevant in vitro model is the type of cells used in the setup. More specifically, whether these are primary cells, cell lines, or induced pluripotent stem cells (which are then differentiated into the desired phenotype), each type of cell has advantages and disadvantages, which are analyzed below.

#### Advantages and disadvantages of cell lines and primary cultures

Animal and human-derived cell lines have been developed as biological surrogates for BBB modeling. One attractive feature of these cells over primary cultures is their relative affordability and (to some extent) their capability of retaining their differentiating properties over multiple passages. Only a few immortalized human endothelial cell lines (HCMEC/D3 [84], HMEC-1 [131], TY08 [132], hBMEC [133], and BB19 [134]) have been developed and reported in BBB modeling. Highly purified populations of cultured human brain (human brain microvascular endothelial *cells*—HBMEC), unfortunately, are quite expensive if acquired from commercial sources. Unfortunately, the high level of technical skill necessary to isolate these cells as well as long term viability after few passages makes their use limited to few laboratories in the field. Isolation of these cells from the native brain tissue also requires advanced technical skills (isolation and purification processes are labor-intensive), time, and a viable source. Human specimens provide features specific to a variety of neurological etiologies that otherwise would be near impossible to recapitulate in cell lines or animal brain-derived (rodent, porcine, or bovine) primary cultures. However, primary cells may provide an attractive alternative in a sophisticated set-up. Regarding Spheroid, hBMEC, pericytes, and astrocytes spontaneously form into a multicellular spheroid in co-culture under low-adherence conditions and self-assemble into a modular organization that resembles the BBB [98]. Needless to say, spheroid provides each cell type to interact with one another, which has been reported to play a pivotal role in the maintenance of BBB integrity and function [99]. In the microfluidic platform, hBMEC might be superior due to its shallow cell requirement.

#### Human-induced pluripotent stem cells (iPSCs) modeling

Even though HBMECs are not yet a realistic (cost-effective) alternative to the use of cell lines for industrial (pharmaceutical) screening (such as testing permeability, toxicity, etc.) and testing of novel drugs, they have unmatched value for basic and translational research. Next to these primary cells, recent advancement in the field of BBB modeling has brought forward the use of human iPSCs [135, 136]. In recent years, with improvements in understanding of differentiation pathways, induced Pluripotent stem cells (iPSCs) are now regularly differentiated to different cell types for co-culture models in microfluidic devices as well as 3D printed models [137]. 3D structure, cell–cell interaction, and the exposure of shear stress result in better barrier function compared to conventional transwell models. iPSCs-based BBB models are the first human BBB models with in vivo like paracellular barrier properties [138]. Thus, these cells may hold enormous potential for the development of preclinical disease models as well as species-dependent differences [139]. The unique advantage of using iPSCs is their ability to generate a representative model of patients from which they have been originated [140]. Additionally, recent advances in gene editing techniques provide exciting opportunities in disease modeling using iPSCs, although these methods still have hindrances like epigenetic reprogramming and loss of patient-specific epigenetic signature [141]. iPSCs technology has some major limitations so that the risk of tumor formation is present due to the use of viral infections and low efficiency of reprogramming during the production of these cells. [142]. On the other hand, an existing limitation of the model is the narrow experimental window provided by iPSC-derived cells, which generally tend to de-differentiate quite rapidly under in vitro culture conditions. Moreover, the cell differential procedure depends upon various random and permanent insertion of transcription factors. Overall, iPSCs- could be applied in the construction of 3D models such as spheroids or organoids in order to support the development of a brain vasculature within these models [139]. Other future 3D models based on iPSCs might use cultivation in or on plastic scaffolds or hydrogels with defined 3D structures. Further development in the field and specifically in the cell differentiation processes and culture stabilization will undoubtedly further the availability and use of these cells in this and other fields of research.

## Conclusion

Over the decades, there has been considerable interest in developing more sophisticated and realistic in vitro models to understand the processes regulating barrier genesis and barrier functions of the BBB and to detect the mechanisms that alter these characteristics under pathological conditions. It is likely to be challenging to find an appropriate model to meet all kinds of experimental requirements and the efficacy of each model is mostly based on the desired downstream assay or translational applications of the researcher including cost, time requirements, ease of setup/operation, and the sensitivity of the findings. Each model system exhibits its unique limitations and thus often requires researchers with a focus on the BBB to utilize multiple platforms for assessment. Although we are still far from mastering this technology, iPSCs cells could indeed deliver a breakthrough in BBB modeling, allowing for the development of the desired cell cultures for the development of organoids systems or simply as a replacement for primary human cultures. In terms of platform development, although the culture-based in vitro BBB models are useful tools to study the transportation and the development of brain-penetrating drugs, reproducibility of BBB properties and function is a significant challenge in these models. It seems that organoids, spheroids, and 3D printed microfluidic systems are enjoying rapid growth, and awareness of this technology among the various laboratories is spreading fast. Although the availability of these platforms is still confined within the laboratories/research groups who are in the early stages of development, the potential for a wide adoption among the scientific community and perhaps industry is increasing. A further boost to the technology could come using multiple techniques in conjunction, such as organoids on a chip where the intrinsic components and structure(s) of the targeted tissue can be reasonably combined in vitro and with high precision.

## Data Availability

Not applicable.

## Abbreviations

BBB: Blood–brain barrier
CNS: Central nervous system
NVU: Neurovascular unit
EC: Endothelial cells
TJ: Tight junction
P-gp: P-glycoprotein
BCRP: Breast cancer resistance protein
MRPs: Multidrug resistance-related proteins
NADPH: Nicotinamide adenine dinucleotide phosphate
TBI: Traumatic brain injury
MS: Multiple sclerosis
HTS: High throughput screening
SS: Shear stress
TEER: Trans-endothelial electrical resistance
DIV: Dynamic in vitro
SOD: Superoxide dismutase
FAD: Familial Alzheimer’s disease
APP: Amyloid precursor protein
PSEN1: Presenilin 1
HD: Huntington’s disease
PD: Parkinson’s disease
MPTP: 1-Methyl-4-phenyl-1,2,3,6-tetrahydropyridine
3D: 3 Dimensional
ROI: Region of interest
iPSC: Induced pluripotent stem cells
FITC: Fluorescein isothiocyanate
IVIVC: In vitro in vivo correlations
VEGF: Vascular endothelial growth factor
MALDI: Matrix-assisted laser desorption/ionization
HBMEC: Human brain microvascular endothelial cells
HBP: Human pericytes
HA: Human astrocytes
HM: Human microglia
HO: Human oligodendrocytes
HN: Human neurons
GLUT: Glucose transporter
ECM: Extracellular matrix
RBE4: Rat brain endothelial cells
CAD: Computer-aided design
ABS: Acrylonitrile butadiene styrene
PLA: Polylactic acid
PA: Polyamide
PC: Polycarbonate
PTM: Printed transfer molding
FDM: Fused deposition modeling
SLA: Stereolithography
SLS: Selective laser sintering
DLP: Digital light processing
LDM: Liquid deposition modeling
FEAM: Fiber encapsulation additive manufacturing
NaFlu: Sodium fluorescein
